# Service of M. Clymer, M. D.

**Published:** 1843-12-23

**Authors:** George N. Burwell

**Affiliations:** Resident Physician


### PHILADELPHIA HOSPITAL.

(Service of Meredith Clymer, M. D.)

Reported by George N. Burwell, M.D., Resident Physician.

### CASE I.

### PNEUMONIA—BRONCHITIS—DIARRHŒA—PREGNANCY—RECOVERY.

Elizabeth R.—aged 27, married, entered the hospital the evening of August 5th. She stated that about a week before, she was taken with a chill after exposure, which was succeeded by a fever and general pain, and considerable dyspnœa; was bled two days after the chill with benefit.

We found her with great heat of skin; profuse perspiration, standing in drops on her forehead; tongue coated with a brownish fur in the middle; red at the edges; pulse 120, full; respiration 54, high and confined to the right side of the chest; cough suppressed; no expectoration; face flushed; dilatation of nostrils. Percussion anteriorly clear, except in the precordial and axillary regions of the left side; posteriorly clear except the upper portion of the left side, and where pain is felt on percussion. Respiration anteriorly clear, except in the axillary region of the left side, where a crepitant rhonchus is heard; posteriorly tubal over a small portion of the lower lobe of the left side, and crepitant rhonchus throughout the same lobe most perceptible in the axillary region. Some nausea and vomiting; epigastrium tender to pressure. ℞. C.C. No. iv. over lower lobe of left side posteriorly. Half a grain of calomel also directed to be taken every two hours. The next morning found her considerably relieved, pulse fallen to 102, and of less volume, respiration 36, with less dysponœa and fever. The nausea continues; bowels open five times. Physical signs as last night. Three cut cups applied to axillary region, and three grains of Pulv. Doveri added to the calomel on account of the diarrhœa.

August 7. General symptoms, pulse, &c., as yesterday. The respiration fallen to 28, with a corresponding change in the physical signs; the pneumonia seems now resolving itself; no dulness on percussion; the crepitant rhonchus changing into a submucous; same sibilant rhonchus heard on the right side; the diarrhœa continues; bowels open nine times since yesterday morning; stools thin, and passed without pain; complains of great nausea, vomited once yesterday; tongue thickly coated with a yellowish fur, red at the edges, papillæ elevated and injected; thirst very great; no appetite, no tenderness of abdomen on pressure; continue the calomel and Dover's powders; gum water for diet; fomentations to the abdomen.

*Evening.*—We were called in haste to our patient at 5 o'clock, this afternoon, on account of a great increase in the diarrhœa, which had become a violent dysentery; we found that she had been constantly on the close stool since two o'clock, complaining of severe and constant cutting pains throughout abdomen and especially over the region of the colon; tenesmus constant and urgent; could not detect any especial tenderness of the belly, "it was all sore;" no tympanitis; icterus (she is in about the fifth month of pregnancy) contracted into a hard mass; stools of a brownish yellow colour, offensive, thin and serous, no blood in them; when in bed lies with the limbs drawn up, and its constantly moaning; ordered forty leeches to be applied over the course of the colon, and followed by a warm flax-seed poultice with a tea spoonful of laudanum in it; gave also an injection of aq. opii. f℥ii. in an ounce of mucilage, to be repeated in an hour.

At 9, P. M. found her lying quite easy with only an occasional pain. Bowels open only twice since 6 o'clock; injection to be repeated after every stool.

August 8. Bowels open five times since last visit; has had no pain since midnight, and no pain now on pressure over abdomen; no nausea; thirst great; skin warm and moist; pulse 110; respiration 28; percussion clear throughout the chest; very little submucous in lungs; same sibilant and sonorous rhonchus in both lungs; cough without expectoration; an infusion of ipecacuanha directed, and warm fomentations over abdomen.

From this time to the date of full convalescence, August 13th, we had to treat principally the diarrhœa, which yielded to the oleaginous mixture. The infusion of ipecacuanha was continued as a diaphoretic and expectorant.

On the evenings of the 9th, 10th and 11th, she had an exacerbation of fever without any local symptoms to account for it; a grain of sulphate of quinia was given every two hours during the 12th, and stopped it.

#### *Remarks.*

This was an admirable case to demonstrate the conversion of pneumonia into bronchitis, and then a return to health. It is doubtful how much was due to the treatment for this change, and how much to the diarrhœa and dysentery; I am inclined to attribute the result, however, to the latter cause.

Another point of interest was the entire absence of expectoration, either during the disease or the convalescence; the cough which she complained of was not at all harrassing.

The effect of the leeches in relieving the acute cutting pains of the dysentery was most happy; in three hours after their application, she was able to lie comfortably in bed, having been the three previous hours scarcely at all in bed, owing to the severity of the pain and the tenesmus. The danger of abortion was great at this time, and required energetic treatment. Fears were entertained that the opium given in the injections, and put upon the poultices, might have a bad influence on the bronchitis, but this it appeared not to do. The strength of the aq. opii was ℥i to the pint, and two and a half fluid ounces if it were given altogether, equal to ten grains of opium, if the water had extracted its full virtues, which it probably did not; of this two fluid ounces, or eight grains were given during the first fourteen hours.

### CASE II.

### FEVER—EXTENSIVE BRONCHITIS, WITH ABSENCE OF ALL RATIONAL SIGNS. INTESTINAL HEMORRHAGY—RECOVERY.

C. S., Irish, aged 23 years, labourer, entered the hospital the evening of September 28th, with general febrile symptoms.

*Detailed note, Sept. 29th.* No satisfactory account can be obtained from him, respecting his sickness, further than it came on three weeks ago, without a chill, but with fever and general illness, severe enough to confine him at once to his bed; he had been at work before this on a coal wharf.

With the exception of a good deal of muttering during his sleep he rested pretty well last night. The expression of his face this morning is dull, and is somewhat flushed; he is easily roused, but answers questions hesitatingly; is much inclined to sleep, has no heat of head nor headache; complains of dizziness and a buzzing in his ears; sense of hearing good; tongue moist with a yellowish fur; a viscid secretion about the mouth and lips; abdomen flat tympanitic, which tympanitis extends to the precordial region, obliterating the natural dulness of percussion observable there, as also the impulsion of the heart; both sounds of this organ can be heard: however; bowels not open since his entrance, tenderness on percussion below the liver; none of the rose-coloured papulæ of typhoid fever observable on the abdomen; pulse 88, regular, and of moderate strength; skin moist; respiration 36, easy and regular, no expression of dyspnœa, no dilatation of nostrils, no cough, no expectoration, no pain; percussion of chest clear anteriorly, quite dull posteriorly over lower lobes; sonorous and sibilant rhonchi heard in both lungs anteriorly and very abundantly; posteriorly these sounds are heard principally in the right lung, mixed with a fine sub-mucous rhonchus, approaching to the crepitant; but little of them in the left lung posteriorly; a distinct fremitus felt on touching any part of the chest; decubitus dorsal. ℞. Six cut cups were applied to the right side posteriorly; a powder of rhubarb and soda given to open the bowels, and a table spoonful of the neutral mixture every two hours. Felt much relieved at evening; pulse 92; respiration 28; the sonorous and sibilant rhonchi much less abundant; bowels opened once; ℞. Four cut cups to axillary right side.

Sept. 30th. Remained in much the same state as yesterday; had a profuse sweat at 7 o'clock this morning, since which his tongue has become dry; pulse rose during the day to 120. ℞. Venesection f℥xiv; ℞. Hydr. c. mit. gr. xii; Kerme's mineral gr. j; Potass nit. ʒss. M. in chart No. vi. divided; one to be given every four hours.

Oct. 1st. Improved to day; face less flushed; pulse 96, small, skin warmer than natural; no uneasiness of head, no muttering during sleep, as on the first night; had some sweating again at seven o'clock this morning; some subsultus; tongue moist; bowels open once since last night; respiration 36; no dyspnœa, cough or expectoration; the amount of the rhonchi in the lungs much less than before, especially in the left; otherwise the physical signs remain as heretofore; continue the powders and give also the neutral mixture every four hours.

Oct. 2d. The general improvement continues; skin hotter than before; no sweating this morning.

Oct. 3d. Much the same; was somewhat delirious last night; tongue dry again; mouth not yet sore.

Oct. 4th. Not as well; tongue remains dry, is swollen; a dark coloured, and very viscid secretion, adheres to the lips and teeth; pulse 100; skin continues hot; when asked how he does, always gives the same answer of "first rate;" still inclined to sleep; breathing heavy and laborious; stupid expression of face; no delirium last night; more rhonchi in the lungs; percussion as before; coughed two or three times this morning for the first time; no expectoration; bowels rather freely open. ℞. Emp. Canth. vi×vi. to right side, kept on six hours, and then applied to the left side, P. M. Both blisters have drawn well, but without producing any very evident amelioration of the symptoms; he has now taken about a drachm of calomel in divided doses, and yet no signs of ptyalism.

Oct. 5th. The obstinacy of the bronchitis, with the observation of the case so far suggesting the opinion that it was secondary to a fever, possibly remittent; it was determined to day, to give the sulphate of quinia freely, commencing early in the morning, as there had been at this time on two days at least, an evident remission; he was this morning, before taking this remedy, in much the condition of yesterday; pulse 100; skin dry, and harsh, but not hot; face rather pale, tongue dry, swollen, sordes about the lips and teeth; expression stupid, physical signs as before. Two grains of the sulphate of quinia were given in solution, every hour; at three o'clock, P.M. his pulse had gone up to 120; skin warm; had taken thus far sixteen grains; take two grains at five o'clock and again at seven o'clock, and stop, which made a scruple in all to-day.

Oct. 6th. Has a much better expression of face, there being but little stupor observable; tongue moist and cleaning; bowels open but once during the night; the stool being thin and brownish; pulse 108, soft; skin keeps dry; no cough, nor expectoration; some sonorous rhonchus heard in both lungs, the most in the upper lobe of the right lung anteriorly; considerable sub-mucous in the lower lobe of right lung posteriorly, almost amounting to a crepitus; percussion dull posteriorly; took but three doses (six grains) of the sulphate of quinia to day; during the rest of the time took neutral mixture; at bed time had his body sponged off with tepid vinegar and water, and gave him a foot bath.

Oct. 7th. Does not appear to be as well as yesterday, tongue a little dry again in the centre; still a tendency to sleep; continue the sulphate of quinia every four hours.

Oct. 11th. Remained in much the same state as before, and under the use of sulphate of quinia, and the neutral mixture; tongue remains moist; last night, the pulse rose from 108 to 118; had a copious stool this morning, with large clots of blood; Hope's nitric acid mixture, without the laudanum, ordered; other medicines discontinued.

Oct. 12th. Much the same; pulse up to 132, less full; give the sulphate of quinia again; arrow-root and beef tea, with wine for diet.

Oct. 13th. The stools became more natural yesterday; tongue much improved; pulse weaker, 130; the rhonchi not materially changed; three grains of the carb. ammoniæ, directed every two hours alternately with the sulphate of quinia; the nitric acid stopped.

Oct. 17th. Continues dull, has had delirium every night lately, gets out of bed; subsultus; had to day a slight epistaxis; respiration 36, puerile anteriorly; sub-mucous rhonchus posteriorly in both lungs, abundant in the right lung, less of it in the left; dulness of percussion posteriorly over lower lobes of both sides; bowels open once this morning, and twice in the night in the bed; stools thin, and yellowish, pulse 128, and of moderate strength; a bad sore forming on the sacrum.

Take the nitric acid mixture as before; wine f℥iv. a day; other medicines discontinued.

Oct. 18th. Intelligence better; begins for the first time to lie on the side on account of the sore, his decubitus having been heretofore invariably on his back; pulse 120; has a moderate diarrhœa.

Oct. 20th. Remains much the same, complains more; cough insignificant; tongue moist, not furred; pulse 120; skin dry; eat some chicken yesterday, with a great relish; has a little delirium every night, passed his stools in bed last night. ℞. Ammon mur. ʒii, spts. etheris nit. f℥ss. aquæ q. s. f℥vi, s. f℥ss. qh. 3. ℞. Pil Hydrarg. gr. v, at night, followed by fʒii, of oil.

Oct. 23d. Diarrhœa better, stools healthier, bowels open three times, in the last twenty four hours. Tongue moist; pallid; pulse 112, moderately full; respiration 32; less rhonchi in chest than at last note; still delirious every night; continues to lie on the side; appetite excellent; complains greatly of pains in his legs; continue the ammonia and nitre.

Oct. 24th. A blister applied to the nucha, for the delirium.

Oct. 26th. Delirium lessened and feels quite well; no rhonchi to be heard in the chest; absence of respiration in the lower lobe of the right lung; vesicular throughout the left lung; pains in the legs continue quite severe. The notes were here discontinued, as the patient was entering upon a decided convalescence; the little diarrhœa soon stopped, and the action of the stomach and bowels, became in every way healthy by the first of November; all medicines were soon discontinued, and a good diet directed; nevertheless, the man remained weak, and it was sometime before he had strength to leave his bed; after this he improved more rapidly.

*Remarks.*

This case was an exceedingly interesting one, in the first place, on account of the entire suppression of any general symptoms indicating inflammation of the lungs, if we except one symptom, the frequency of respiration. He had neither pain, nor dyspnœa, nor cough nor expectoration. The occasional cough was more referable to the condition of the mouth and throat, than to that of the lungs. There was no expectoration during his sickness, although during the greater part of the time, there existed an abundant sub-mucous rhonchus. It will be seen that this is very like the first case reported, where the same condition of things existed. Such cases are occasionally met with and are exceptions to the general rule, that bronchitis relieves itself by expectoration. I cannot say that these cases were in any way prolonged on this account.

This was an interesting case, in the second place on account of the complication of extensive bronchitis with a fever. He had been sick three weeks, and was brought in probably about the time of the supervention of the bronchitis. This was treated exclusively for the first few days, the idea of the complication not suggesting itself. As soon as the bronchitis yielded a little, the sulphate of quinia, was given freely for one day, in the hopes of producing a decided result, while these effects were closely watched. The raising of the pulse, which followed, was not well marked until 3 P. M., the first dose having been given at 3 A. M. The man's improvement the next day was perceptible, especially in the clearing of the tongue and in the cerebral symptoms.

Was the fever typhoid or remittent? This is a question about which there must remain considerable doubt, and is the most important point connected with the case. The season of the year, the remission he had on two or three successive mornings, would favour the idea that it was remittent fever; while the early and continued dulness of the mind, and other cerebral symptoms, the epistaxis towards the close of the disease, the diarrhœa, and discharges of blood from the bowels, and even the bronchitis itself, belonged rather to typhoid fever. The effects of the sulphate of quinia would also encourage this conclusion; for the improvement in the fever was not positive enough for remittent fever. The abdomen remained flat throughout his illness, although there was some tympanitis, and without tenderness, except at first, over the liver. There were no sudamina, nor the papular eruption of typhoid fever.

The case next reported occurred at the same time, in the women's medical wards, and gave the same difficulty in diagnosis.

### CASE III.

### TYPHOID FEVER—UTERINE HEMORRHAGE—DEATH—AUTOPSY—REMARKS.

F. M., a German, aged forty years, entered the medical wards Sept. 24. I saw her that afternoon with Dr. Cary, whose patient she was. She could not speak English, and besides, was too dull to give any satisfactory account of herself. We could only learn that she had had a child about six weeks before, was intemperate, and had been exposed to the weather of late. She complained of being sick all over. Pulse 120, small, and rather hard; skin dry and warm; face flushed, and had a dull, heavy, livid tint; expression of face and eyes stupid, unless roused, when her eyes would assume a much brighter and more natural expression; has some subsultus; lies with her mouth partly open; tongue dry and brown, not furred, somewhat glazed; bowels open once a-day; belly slightly tympanitic and swollen, and quite tender on very moderate pressure; the femoral veins tender also, but not swollen; could not detect any enlargement of the liver or spleen; no vomiting; no typhoid papulæ or sudamina. Respiration natural; no cough or uneasiness of the chest. On examination, per vaginam, the os uteri was found quite dilated, and rather tender on pressure. The right heart sore and swollen. Small doses of blue pill, and the neutral mixture directed.

25. In much the same state. Under the idea that it might be a case of low form of remittent fever, and there being no symptom forbidding it, a grain of the sulphate of quinia was given every hour. The blue pill continued.

26. There was something of a remission. The pulse fell to 108. No increase of the tympanitis; the tenderness of the abdomen continued, was general, but most marked in the iliac and hypogastric regions.

27. A tendency to diarrhœa, which was checked by an injection of fifteen drops of laudanum. Poultices to the abdomen.

28. Quite a tendency to prostration; bowels not open since the injection yesterday; no thoracic symptoms; fever subsiding; less flush of the face; expression dull; tint of face livid; tongue continues dry; moans out quite loud at intervals. Ordered wine.

29. Prostration continues; bowels not yet open; no more moaning; commenced flooding to-day; the discharge comes on at intervals with a gush, and is very free; other symptoms as yesterday. Directed some oil. The poultices to abdomen discontinued. ℞. Ammon. Carb. gr. v., q. h. 2.

30. Rallied some last night and this morning. The other symptoms no better.

Oct. 1. The stupor increased during yesterday; was with difficulty roused last night; this morning perfectly insensible and moribund. Died at 6, P. M.

*Post-mortem forty hours after Death.*

Lungs healthy. Liver not enlarged, but much softened, and red. Spleen of a dark blue colour, and somewhat enlarged—not notably softened. Mesenteric glands enlarged and softened. The subperitoneal coat of the intestines so softened that they could be stripped out from the peritoneum. Considerable injection of the mucous membranes of the intestines, both large and small, and in portions deeply and fine enough to constitute inflammation. This was particularly noticed at the head of the colon, and in different portions of the ilium, corresponding to a number (eight or ten) of enlarged, elevated, and injected patches of the glands of Payer. The lowest of the patches was situated about two feet above the ileo-colic valve, and thence extended at intervals up to the central and upper portions of the ilium. These patches were in no instance ulcerated.

*Remarks.*

This was an undoubted case of typhoid fever, and in many respects it resembled the case of C. S., especially in the non-app arance of the external symptoms so generally looked upon as diagnostic. In neither case did the cerebral symptoms appear grave enough at first to say positively that it was typhoid fever, when taken in connection with the absence of the cutaneous eruption, and the slight abdominal symptoms existing. No absolute diagnosis between remittent and typhoid fever could be made, therefore, at the commencement in either case; and it became a mere calculation of probabilities, in the decision, as to which it was.

In the man's case, the discharge of blood from the bowels, the epistaxis, the increase of the delirium, his attempts to leave his bed at night, the diarrhœa and involuntary stools together, served to clear up the diagnosis, and to render it quite positive. The pains in the legs were quite severe, more so than ordinary in those cases of convalescence from typhoid fever in which they are met with.

The points worthy of observation in the woman's case are, *first*, her age, forty years, greatly above the average age in which we meet with this fever; *second*, the great tenderness of the abdomen; *third*, the occurrence of uterine hemorrhage two days before death; and *fourth*, as already mentioned, the absence of the typhoid or cutaneous eruption.

